# Monoacylglycerol Form of Omega-3s Improves Its Bioavailability in Humans Compared to Other Forms

**DOI:** 10.3390/nu12041014

**Published:** 2020-04-07

**Authors:** Bernard Cuenoud, Isabelle Rochat, Maria Laura Gosoniu, Lenaick Dupuis, Evan Berk, Anke Jaudszus, Jochen G. Mainz, Gaudenz Hafen, Maurice Beaumont, Cristina Cruz-Hernandez

**Affiliations:** 1 Nestlé Health Science, Translation Research, Avenue Nestlé 55, CH-1800 Vevey, Switzerland; Bernard.Cuenoud@nestle.com; 2 Department of Pediatrics, Respiratory Unit, Centre Hospitalier Universitaire Vaudois, CH-1005 Lausanne, Switzerland; irochat@hotmail.com (I.R.); dr.hafen@hin.ch (G.H.); 3 Nestlé Research, Sociéte des Produits Nestlé SA, Lausanne, Vers-chez-les-Blanc, PO Box 94, CH-1000 Lausanne 26, Switzerland; MariaLaura.Gosoniu@rdls.nestle.com (M.L.G.); Lenaick.Dupuis@rdls.nestle.com (L.D.); mauricegillesbeaumont@gmail.com (M.B.); 4 Nestle Health Science, ‘Metabolic Health’, Bridgewater, NJ 1041, USA; Evan.Berk@US.nestle.com; 5 Pediatric Pulmonology Section, Cystic Fibrosis Centre for children and adults, Jena University Hospital, 07747 Jena, Germany; Anke.Jaudszus@med.uni-jena.de (A.J.); j.mainz@klinikum-brandenburg.de (J.G.M.); 6 Department of Pediatric Pulmonology/Cystic Fibrosis Center, Brandenburg Medical School (MHB) University, 14770 Brandenburg an der Havel, Germany

**Keywords:** omega-3 *sn*-1(3)-monoacylglycerol, fatty acid digestion, omega 3, lipid absorption, cystic fibrosis, obesity, hypertriglyceridemia

## Abstract

Numerous benefits are attributed to omega-3 fatty acids (OM3) especially in cardiovascular health. However, bioavailability and clinical efficacy depend on numerous factors, including OM3 form, food matrix effects (especially the lipid content of the diet), and metabolic capacity. Here, we show in humans that a “pre-digested” OM3-*sn*-1(3)-monoacylglycerol lipid structure (OM3-MAG) has a significantly greater absorption at high therapeutic doses (2.9 g/day) than the most commonly OM3-ethyl ester (3.1 g/day) form (used for the treatment of hypertriglyceridemia), and a comparable profile to other pre-digested OM3 free fatty acids (OM3-FFA) structure (3.2 g/day). Nutritional supplement doses of MAG resulted in similar increases in OM3 blood level, compared to OM3 triacylglycerols (OM3-TAG) supplements in obese subjects (1.2 g/day) under low fat diet, and in children with cystic fibrosis (1.0 g/day). These results suggest that both forms of pre-digested OM3-MAG and OM3-FFA are effectively absorbed and re-incorporated effectively into triacylglycerols inside the enterocytes, before being exported into the chylomicrons lipid transport system. The pre-digested OM3-MAG might provide a more effective therapy in severe cardiovascular conditions where high doses of OM3 are required and a low-fat diet is indicated, which limited digestive lipase activity.

## 1. Introduction

Omega-3 fatty acids (OM3) such as eicosapentaenoic acid (EPA) and docosahexaenoic acid (DHA) have pleiotropic effects in supporting health and improving diseases [1,2], most notably by reducing cardiac death from major cardiovascular disease events, helping hypercholesterolaemic patients [3,4,5,6,7,8,9,10]. The key mechanisms involved include (1) reducing inflammatory processes by impairing the activation of a key inflammatory regulator, NF-κβ, (2) altering the expression of genes that regulate fat metabolism, (3) upregulating FA oxidation, and downregulating lipogenesis in fat tissue [6,7,8,11,12,13].

**Figure 1:**
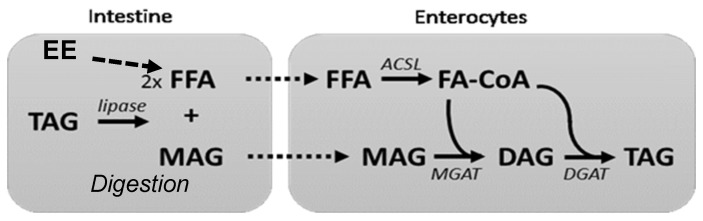
Digestion of fat and re-synthesis in the enterocyte for efficient chylomicron formation and systemic absorption in erythrocytes. Ethyl ester (EE), acyl-CoA synthetase long-chain (ACSL), Di-Acyl Glycerol (DAG), Monoacylglycerol acyltransferase (MGAT), Diglyceride acyltransferase (DGAT); Fatty Acid CoA (FA-CoA).

During digestion, lipolysis of dietary fat releases two free fatty acid (FFA), and *sn*-2 monoacylglycerol (*sn*-2 MAG); followed by the formation of micelles, which enhance and facilitate bioavailability [14,15]. FAs at the *sn*-2 position are absorbed directly as MAG (Figure 1). The endogenous conversion rate of the essential fatty acid α-linolenic acid into EPA and further to DHA is limited in humans [14,15,16]. Therefore, it is recommended to include sources of OM3- long chain polyunsaturated fatty acids (LC-PUFAs), such as fatty fish, in one’s diet in order to obtain the desired health benefits [15,17,18,19].

Several bioavailability studies have been devoted to the actions of different sources of LC-PUFAs (e.g., fatty fish versus supplements) or different carriers (e.g., triacylglycerol (TAG), MAG, ethyl ester, FFA, and phospholipids) [5,6,12,20,21,22,23]. During the digestion of the ethyl ester carrier, the ethyl group is hydrolyzed by lipases, and the resulting fatty acid (FA) is then converted back into a TAG molecule (Figure 1) before being transported by the lymphatic system [24,25]. Some studies have shown that this process is less efficient because the pancreatic lipase hydrolyzes ethyl ester slower than it does TAG, and the lack of the *sn*-2 MAG backbone from the TAG structure that is necessary for TAG resynthesis within enterocytes [14,24,25,26,27,28].

**Figure 2:**
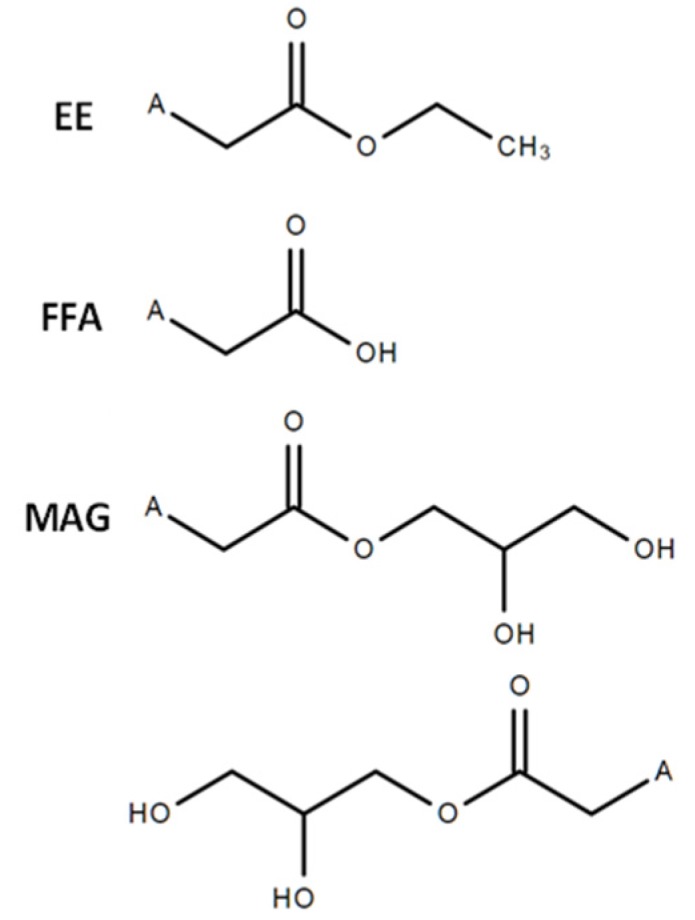
The chemical structures of the OM3: ethyl ester, free fatty acid and sn-1(3)- monoacylglycerol products. A = fatty acid (e.g., EPA, DHA).

Besides the general health claims, high doses of OM3 have been developed as drugs for patients with hypertriglyceridemia [2]. The first prescription OM3 drug in the form of an ethyl ester (Figure 2) as EPA and DHA (1.4–3.4 g daily doses) was approved (Lovaza^®^;GlaxoSmithKline, Research Triangle Park, NC, USA) and indicated as an adjunct to the diet to reduce TAG levels in adult patients with hypertriglyceridemia (>500 mg/dL) levels. Subsequently, FFA (Epanova^®^, AstraZeneca, Cambridge, UK) and an EPA- ethyl ester (Vascepa^®^, Amarin Pharma, Inc. Bridgewater, NJ, USA) OM3 forms were approved with the same indication. The REDUCE-IT clinical study (Reduction of Cardiovascular Events with Icosapent Ethyl-Intervention Trial) demonstrated that 4g of EPA ethyl esters could decrease the risk of major ischemic events, including a 20% reduction in cardiovascular death, in patients with elevated TAG level despite being on statin therapy [29].

In its 2019 session, the Science Advisory from the American Heart Association concluded that the prescription OM3 either as EPA + DHA or EPA alone (at a dose of 4 g/day) are an effective and safe option for reducing TAG as monotheraphy or as an adjunct to other lipid-lowering agents [30]. The OM3 LC-PUFAs as FFA form has been shown to be better bioavailable than those in TAG or ethyl ester forms [7,27,31,32], likely because FFA does not require enzymatic hydrolysis for absorption [27,28]. Studies comparing FFA versus ethyl ester suggested also that there are differences in bioavailability based on fat diet content. In subjects on the low-fat diet, the FFA had higher bioavailability than the ethyl ester form [25,26,27].

Defects in either intraluminal fat digestion or uptake and transport of its digestive products across the gut barrier may lead to fat malabsorption and/or maldigestion [33,34]. An example of the above is cystic fibrosis, a genetic disorder affecting multiple organ systems that also exhibits exocrine pancreatic insufficiency. Cystic fibrosis patients using enzyme replacement therapy still show a certain degree of steatorrhea, which indicates additional underlying causes of maldigestion. Indeed, it has been suggested that this type of persistent malabsorption is not only due to insufficient enzymes but also due to incomplete intraluminal solubilization or reduced enterocyte uptake, or both [33,35,36]. Studies suggest that OM3 LC-PUFA may be anti-inflammatory and a benefit for cystic fibrosis patients, and that the MAG form exhibits a higher degree of efficient absorption than the TAG form [20,21,22,23,28,33,35,36].

Recent studies evaluating the bioavailability of MAG containing EPA/DHA have found that this carrier exhibits a high degree of efficient absorption [23,28,32]. Also, studies have reported that FAs at the *sn*-2 position are more efficiently absorbed by the intestine. However, a challenge with *sn*-2 structures is that they are not very stable and easily undergo isomerization to form *sn*-1-(3)-MAG structures. Interestingly, OM3 was shown to be an effective substrate to MGAT enzyme and could be used as a pre-digested fat [37,38] with potentially improved bioavailability compared to ethyl ester or TAG. This lipase-independent MAG carrier may offer a clinical advantage to patients struggling with fat malabsorption/maldigestion problems, for instance, patients with cystic fibrosis [21,37,38,39].

In this report, we explore the bioavailability of OM3-MAG in humans with different metabolic capacity, and how it compared with other forms of OM3. In our first study (clinical trial A), we aimed to demonstrate that *sn*-1(3)-monoacylglycerol oil (OM3-MAG) might be more bioavailable when compared to ethyl ester and FFA carriers in healthy subjects under low fat diet. Second, the bioavailability of the MAG was compared to TAG oil in overweight/obese healthy subjects under a low-fat diet by measuring DHA/EPA levels in blood plasma (clinical trial B). Third, the bioavailabilities of the MAG and TAG oils were compared in cystic fibrosis patients with known exocrine pancreatic insufficiency. The absorption of LC-PUFA was compared by the measurement of DHA and EPA in plasma and erythrocytes (clinical trial C). The different study groups and clinical trials are summarized in Table 1.

## 2. Materials and Methods 

### 2.1. Clinical Trial Ethics

All clinical trials were conducted in line with the guidelines in the Declaration of Helsinki and study protocols were approved by the Commission cantonale d’éthique pour la recherche sur l’être humain (Ethics Committee of Lausanne Switzerland) and the Institutional Review board IntergReview, Austin, TX. The trials were registered at ClinicalTrials.gov, with the identifier # NCT03017651 for Clinical trial A, # NCT 03,118,999 for Clinical trial B and # NCT02646995 for Clinical trial C.

### 2.2. Subjects

In clinical trial A, healthy adults were recruited and enrolled in a three-arm crossover design. This was a randomized, controlled, acute, open trial. Volunteers were screened at the clinic for dietary and clinical evaluation, anthropometric measurements (age, body mass index (BMI), height, and weight), and verified for exclusion/inclusion criteria (Table 2).

In Clinical trial B, obese or overweight subjects were recruited and enrolled in a two-arm crossover design, randomized, acute, single blind, pharmacokinetic (PK) study. Once the volunteers agreed and/or the informed consent letter was signed, volunteers were verified for inclusion/exclusion criteria (Table 2), subjects were exposed to a complete physical examination, a 12-lead electrocardiogram, an OM3 food frequency questionnaire, and urine pregnancy test. Additional routine clinical examinations (hematology, chemistry, urine analysis) were carried out along with measurement of vital signs and anthropometric measurements (i.e., age, body mass index-BMI, height, and weight).

**Figure 3:**
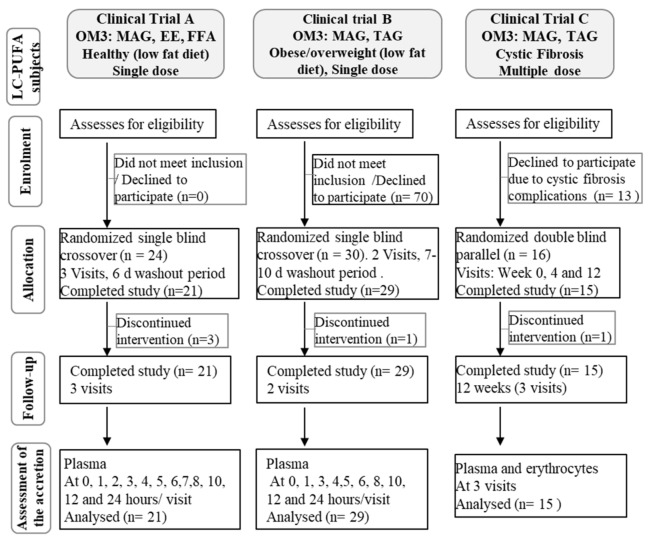
Flow diagram for study participation in clinical trials.

In Clinical trial C, cystic fibrosis patients with exocrine pancreatic insufficiency, were recruited and enrolled in a two-arm parallel, randomized, controlled trial, chronic (12 weeks), double blind for the tested encapsulated oils (Figure 3). Once the volunteers agreed and/or the informed consent letter was signed and dated by minors’ parents or legal guardians, volunteers were verified for inclusion/exclusion criteria (Table 2), subjects were exposed to routine medical and physical examination. Additional screening evaluation included recording of demographic information and a medical history review.

### 2.3. Interventions

In clinical trial A, oil capsules contained either the enriched OM3-MAG oil (Pronova/BASF, Ludwigshafen, Germany) mainly as *sn*-1(3)-monoacylglycerol, OM3-ethyl ester (Omacor^®^, Pierre Fabre, Paris, France), or OM3-FFA (Pronova/BASF, Ludwigshafen, Germany). Capsuled were 1 g, containing mainly EPA and DHA as shown in Table 1 and Table 3.

The OM3-TAG and OM3-MAG oil capsules were provided in the clinical trials B and C. Oils used were provided either as enriched OM3-MAG oil, mainly as *sn*-1(3)-monoacylglycerol (Pronova/BASF, Ludwigshafen, Germany); or as fish oil, mainly as OM3-TAG (Sofinol S.A., Manno, Switzerland). Oils were encapsulated (380 mg oil/capsule) containing 3600 mg/kg of the natural mixed tocopherols as antioxidants (Table 1 and Table 3).

### 2.4. Study Design

Clinical trial A with normal weight subjects was a randomized, controlled, open trial conducted at Nestle Clinical Development Unit in Lausanne, Switzerland. The study followed a three-arm crossover design with a 6-day wash out period between two periods. The three study groups were as follows: (1) OM3-MAG, (2) OM3- ethyl ester, (3) OM3-FFA (Table 1). Each product was given once per day at a single dose. According to the oil product, 4 to 5 capsules were given to the subject as shown in Table 1.

Clinical trial B with overweight/obese subjects was a randomized, single-blind trial conducted at Miami Research Associates, Miami, FL, USA. The trial followed a two-arm crossover design with a 7–10 days washout period between two periods. The two study groups were as follows: (1) OM3-TAG and (2) OM3-MAG (Table 1). Each subject received 9 capsules of study product on day 1 after an overnight fast.

Clinical trial C with cystic fibrosis patients was a randomized, controlled trial conducted at Centre Hospitalier Universitaire Vaudois (CHUV) in Lausanne, Switzerland. The trial followed a two-arm parallel design with double-blinded identity of the tested encapsulated oils. The two study groups were as follows: (1) OM3-TAG and (2) OM3-MAG (Table 1). Subjects ingested 4 (5–10 years old patients) or 8 capsules (11–18- year old patients) per day, for 12 weeks. Doses were distributed at each visit, and compliance was monitored by accounting the returned capsules at each subject visit.

### 2.5. Assessment of Accretion

In clinical trial A, an acute phase to assess kinetics was performed. During visit 1, blood samples were taken from fasting subjects; this was considered the baseline. Following blood collection, subjects were given 4 or 5 oil capsules of the first testing product (Table 1). Blood sampling was performed every hour until 8 h and after at 10 and 12 h (Figure 3). Fat free lunch and dinner were provided at 4 h and 10 h respectively. At visit 2, blood sampling was performed in fasting conditions (24 h) and a standard breakfast was provided. Water intake was allowed *ad libitum* from 0 to 24 h. At visit 3-4 and visits 5-6 the second and third testing products were provided respectively. The three different test periods were separated by a washout period of 6 days. Blood samples were fractionated for the plasma to be analyzed for FA analysis (Figure 3).

In clinical trial B, an acute phase to assess kinetics was performed. Standardized low-fat meals were served on day -1. Meal schedule was similar for visit 1 except no breakfast was provided and lunch was served at approximately 4 h post-dose. At the first visit, blood was drawn after an overnight fast; this sampling point was considered the baseline of the study. Immediately after the baseline was taken, subjects ingested 9 capsules (Table 1). For PK, blood sampling was performed every hour until 8 h and after at 10 and 12 h after capsule intake (Figure 3). Subjects received a standardized low-fat lunch, low-fat dinner, and low-fat snack at approximately 4 h, 10 h and 14 h respectively, after capsules intake. Water intake was allowed *ad libitum*. Next visit, blood sampling was performed in fasting conditions (24 h) and standard breakfast was provided. There was a 7–10 day washout period between product administrations. Plasma was obtained from blood samples to be analyzed for FA analysis (Figure 3).

Biochemical assessments included the blood collection at screening after overnight fast for hematology, serum chemistry: electrolytes (Na and K) glucose, urea, creatinine, albumin, lipid profile (total cholesterol, TC; low-density lipoprotein-cholesterol, LDL-C; high-density lipoprotein-cholesterol HDL-C and TAG), L-aspartate aminotransferase, L-alanine aminotransferase, total bilirubin and urinalysis (pH, specific gravity, protein, glucose and blood). Also, serology, breath alcohol screen, and urine pregnancy tests were performed. Subjects had urine samples collected for a drug and alcohol screen.

In clinical trial C, at the first visit, each subject was instructed to orally consume 4 capsules (8 capsules for patients older than 10 years old) with a glass of water distributed after the breakfast, lunch, and dinner for 12 weeks. Fasting blood samples were taken at baseline, week 4, and week 12 to collect erythrocytes and plasma samples for FA analysis (Figure 3).

At study start, a complete blood count was determined after overnight fast before subjects began the intervention. At first and last visits, lung function (lung clearance index -LCI, forced expiratory vital capacity -FEV1, forced expiratory volume per second -FVC and mean exploratory flow between 25% and 75% of vital capacity -MEF 25/75), nasal lavage (for pro-inflammatory markers measurement and respiratory bacteriology testing), and colonization with *Pseudomonas aeruginosa* (an opportunistic pathogen which triggers progressive lung destruction in cystic fibrosis) in sputum were assessed. Evaluation of inflammation by measurement of cytokines (Interleukine (IL) 1B, IL-6, IL-8, Interferon-γ inducible protein (IP-10), Polymorphonuclear leukocyte elastase (NE) in nasal lavage fluid was also performed as previously reported [40].

### 2.6. Analysis of the Fatty Acid Composition in Blood Lipids

Blood was collected in 1.2 mL (4 mL for clinical trial A and B) Ethylenediaminetetraacetic acid (EDTA)-containing S-Monovette K3 (Sarstedt # 02.1066.001). Plasma was separated from erythrocytes by single centrifugation as previously applied [21,41], no further centrifugation was applied to the erythrocytes fraction obtained [42]. Sample preparation for fatty acid methyl esters (FAME) analysis was done as previously described [21]. Blood fractions were kept on ice during sample preparation and finally stored at −80 °C until FA analysis. As previously described [21,41] FAMEs of erythrocytes and plasma were prepared directly in the tubes in which they were aliquoted and stored. Internal standards FAME 21:0 (1 mg/mL) and phosphatidylcholine 23:0 (0.4 mg/mL) or TAG 13:0 (0.1mg/mL) were added (100 µL each) plus 2 mL of methanol, 2 mL of catalyst methanol/HCl (3N) and 1 mL of n-hexane.

Analysis of total FAMEs was performed by Fast Gas Chromatography (GC), as previously described [21] on a 7890 Agilent gas chromatograph (Agilent Technologies, Palo-Alto, CA, USA), equipped with a fused-silica BPX-70 capillary column (10 m, 0.1 mm i.d., 0.2-µm film thickness; Spencer Group Engineering, Melbourne, Australia).

### 2.7. Statistical Analysis

For clinical trial A, the EPA and DHA doses slightly differ among the products, therefore, to have comparable results between the products, a linear relationship between the dose and the response was assumed. The primary outcome was the baseline adjusted area under the curve (AUC0-24h). AUCs and Cmax values were adjusted to the nominal dose of EPA and DHA provided to the subjects, based on the determination of the exact amount of EPA-FFA and DHA-FFA equivalent contained in the 1g capsules. The primary outcome was analyzed using linear mixed models adjusting for baseline values. The product and the visit were considered as covariates. In addition, the sequence was considered as an independent variable in the model to test for possible carryover effect. No statistically significant sequence effect was observed; therefore, the sequence was not considered in the final model. Subject-specific random effects were added to the model to take the correlation between visits measurements into account. For Tmax, the non-parametric Exact Wilcoxon rank-sum was used with the associated Hodges-Lehmann estimator for the estimated treatment effects, their 95% CI and the associated *p*-value.

For clinical trial B, each pharmacokinetic parameter was summarized (n, mean, median, standard deviation, min, max, logarithmic/geometric mean) by product. PK analyses of baseline-corrected plasma EPA and DHA concentration-time data were conducted by non-compartmental model of Phoenix WinNonlin version 6.3 (Pharsight Corporation, St. Louis, MO, USA). Actual elapsed times from dosing were used to estimate all individual plasma PK parameters for evaluable subjects.

The endpoints of interest were the baseline-adjusted Cmax and AUCs for EPA and DHA which are measured in μg/mL and h·μg/mL, respectively. The ANOVA was estimated at alpha 2.5% on the log-transformed data for Cmax, AUC0-t and AUC0-inf. The ANOVA model included sequence, treatment, and period as fixed effects and subject (sequence) as random effect. The significance of the sequence effect at alpha 5.0% was calculated using the subject nested within the sequence as the error term. The Tmax was analyzed using Non-parametric Wilcoxon’s Rank Sum test. The ratio of geometric least-square means (GMR) of investigational and comparator products was calculated using the least square means (LSMs) for log-transformed pharmacokinetic parameters of Cmax, AUC0-t and AUC0-inf. The geometric mean was reported for the log-transformed pharmacokinetic parameters of Cmax, AUC0-t and AUC0-inf of MAG and TAG oils. 95% two-sided confidence intervals for the difference between treatments least-square means was calculated for the log-transformed Cmax, AUC0-t and AUC0-inf. The confidence interval was expressed as percentages relative to the least-square means of the reference treatment. The success of the trial, superiority of MAG compared to TAG, was concluded if the lower bounds of the 95% confidence intervals for AUC0-t was bigger than the value 1.

In clinical trial C, the primary outcome was the EPA in erythrocytes expressed in units of percent of total FA and mg/dL at 12 weeks. EPA in erythrocytes was approximately log-normally distributed; therefore, a log-transformation was applied. EPA in erythrocytes at 12 weeks was analyzed by Analysis of covariance (ANCOVA) correcting for EPA in erythrocytes at baseline. The treatment difference was displayed in percent with respect to the grand mean at 12 weeks. The key secondary outcome analyses consisted on the lung function and inflammatory markers analysis. Lung-function was captured by the LCI presented by two numbers: The absolute measure and percentage with respect to a norm, which is again a percentage (2.5%). An LCI was assumed approximately normally distributed and the same statistical analysis method was applied as for the EPA in erythrocytes. For nasal lavage inflammatory parameters, normality/ANCOVA assumptions of the statistical model were unmet. Therefore, data was log transformed and product effect was estimated using Wilcoxon rank sum test where the product groups were compared at baseline, study end, and any change during this period.

## 3. Results

### 3.1. Clinical Trial A. Normal Weight Healthy Subjects with Low Fat Diet

#### 3.1.1. Clinical and Compliance Evaluation

Twenty-four healthy adult subjects (9 males and 15 females) were screened and recruited, with an average age of 40 years (female 37 years ± 7.34 and male 41 years ± 8,36) and BMI average of 23 kg/m^2^ (female 22 kg/m^2^ ± 2.04 and male 24 kg/m^2^ ± 1.89). Three subjects discontinued participation voluntarily at visit 1 and 2, time 0. All other subjects were 100% compliant with the study supplements for the duration of their participation in the study.

The provided supplements were generally well tolerated. There were no serious adverse events (SAEs) or other significant adverse events (AEs) observed or reported in this study. There were twenty-two treatment-emergent adverse events (TEAEs) reported by eleven subjects. All AEs were mild in severity, which included nausea, diarrhea, and headache. AEs were considered to likely be related to study product or procedure.

#### 3.1.2. Incorporation of EPA and DHA in Plasma-Acute Phase

The primary objective of this clinical trial was to assess the global absorption of total plasma EPA + DHA following the ingestion of OM3-MAG, OM3-ethyl ester and FFA (Table 1, Figure 3).

At each visit (and each product) the baseline values of EPA (64.6, 68.8 and 69.8 nmol/mL respectively), DHA (153.0, 152.5, 166.6 nmol/mL) and EPA+DHA (217.7, 221.2, 236.4 nmol/mL) levels in plasma were not significantly different.

On the first day, eleven blood samples were taken from 0 to 12 h and a last sample at 24 h in order to measure how EPA and DHA were incorporated in plasma by OM3-MAG, OM3-FFA or OM3-ethyl ester oils intake.

**Figure 4:**
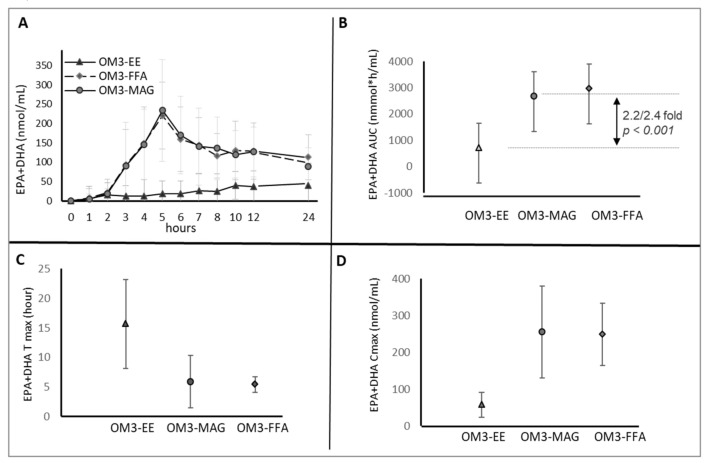
Clinical trial A. Acute effect: Pharmacokinetic results (baseline-adjusted), EPA + DHA in Plasma, AUC over 24 h postprandial (upper panel (**A**,**B**)). Results are expressed in nmol·h/mL. Tmax (hour) and C max (nmol/mL), lower panel (**C**,**D**).

Absorption of OM3-MAG was better than that of OM3-ethyl ester and resulted in higher AUC0-24 h of EPA + DHA (2692 vs. 725 AUC in nmol·h/mL). The linear mixed model showed a product difference of 1912 nmol·h/mL (95% CI 1265–2559 nmol·h/mL, *p* < 0.001) (Figure 4A) between the OM3-MAG and OM3-ethyl ester for AUC0-24 h EPA + DHA. EPA + DHA AUC0-24 h (Figure 4A) after OM3-FFA oil intake was also higher (2852 vs. 725 AUC in nmol·h/mL), with an estimated difference of 2228 nmol·h/mL (95% CI 1560-2896 nmol·h/mL, *p* < 0.001) when compared with OM3-ethyl ester.

An effect modification was observed through time where OM3-MAG and OM3-FFA increased the global absorption of total plasma EPA+DHA compared to ethyl ester. EPA + DHA AUC0-24 h of MAG was 2.2-higher (Figure 4B) when compared with ethyl ester (*p* < 0.001) and 2.4-higher when OM3-FFA and OM3-ethyl ester were compared (*p* < 0.001). A decrease of 0.92 was observed when OM3-MAG and OM3-FFA were compared, although not significant (*p* = 0.36). Significant differences were found in Tmax (Figure 4C) with OM3-MAG and OM3-FFA when compared with the Tmax of ethyl ester group. The EPA+DHA baseline-adjusted Tmax was reached 7 h before, with OM3-MAG and OM3-FFA compared to ethyl ester (*p* < 0.001). No effect modification of EPA+DHA levels between OM3-MAG and OM3-FFA (*p* = 0.79) was demonstrated for Tmax. Cmax demonstrated similar findings to AUC as shown in Figure 4D. Cmax of EPA+DHA of OM3-MAG increased 3.89- when compared with OM3-ethyl ester (*p* < 0.001) and 3.70-increased when compared OM3-FFA and OM3-ethyl ester (*p* < 0.001). The OM3-MAG enriched oil showed a 1.04- increase when compared with OM3-FFA enriched oil, but it was not significantly different (*p* = 0.70).

In general, the same trend that was observed for EPA+DHA was observed for each FA individually. Increases for EPA and DHA were observed in plasma, although the increase in DHA was lower, as is reported in Table 4. The OM3-MAG and OM3-FFA oils significantly increase the bioavailability (baseline-adjusted AUC and Cmax) of total EPA and DHA when compared to the ethyl ester oil. Statistically significant differences were also observed for the time required to reach maximal concentration Tmax (earlier Tmax). There were no statistical significant differences between OM3-MAG and OM3-FFA in terms of incorporation of FA in plasma.

### 3.2. Clinical Trial B. Obese or Overweight Subjects

#### 3.2.1. Clinical and Compliance Evaluation

Thirty healthy adult subjects (12 males and 18 females) were screened and recruited, with an average age of 44.1 years, and group characteristics are shown in Table 5. No significant differences were found among participants regarding total cholesterol, HDL-C, LDL-C or TAG, measured at baseline as shown in Table 5.

One subject discontinued participation voluntarily at visit 1, hence the subject did not receive product in second period and was excluded from the PK population. All other subjects were 100% compliant with the study supplements for the duration of their participation in the study.

The provided supplements were generally well tolerated. There were no serious AEs or other significant AEs observed or reported in this study. There were three TEAEs reported by three subjects. All AEs were mild in severity. One AE (headache) was considered to likely be related to study product. There was one AE (headache) that was considered unrelated to the oral administration of the OM3-MAG test product and another AE (headache) that was considered unrelated to the oral administration of the OM3-TAG test product.

#### 3.2.2. Incorporation of EPA and DHA in Plasma-Acute Phase

The primary objective of this clinical trial was to assess the bioavailability of plasma EPA and DHA following the ingestion of OM3-MAG and OM3-TAG (Table 1 and Table 3, Figure 3).

At baseline EPA levels in plasma were 13.2 and 12.3 µg/mL from OM3-MAG and OM3-TAG products respectively. DHA levels were 52.1 µg/mL and 50.6 µg/mL from OM3-MAG and OM3-TAG products respectively. No statistically significant differences were observed between the two products.

On the first day, nine blood samples were taken from 0 to 8 h and after at 10 and 12 h and a last sample taken at 24 h, in order to measure how EPA/DHA were incorporated in plasma by OM3-MAG and OM3-TAG oils intake.

**Figure 5:**
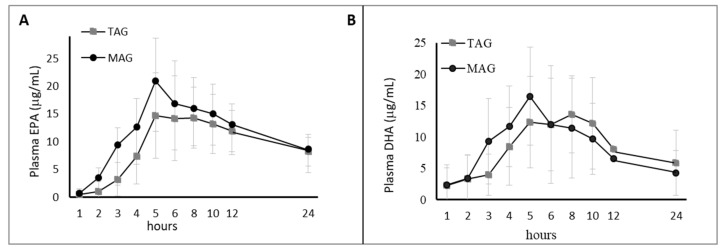
Clinical trial B. Acute effect: Pharmacokinetic results (baseline-adjusted), EPA (**A**) + DHA (**B**) in Plasma, AUC over 24 h postprandial (upper panel). Results are expressed in ug·h/mL.

Uptake of OM3-MAG was better than that of OM3-TAG as shown by the geometric least-square mean ratios (GMRs) of AUC0-24h for baseline corrected EPA between OM3-MAG and OM3-TAG of 116.47% (95% CI: 102.82%, 131.94%), indicating an increase of 16% (Figure 5).

An effect modification was observed over time where OM3-MAG statistically significantly increased the global absorption of total plasma EPA compared to TAG in terms of baseline-adjusted AUC. Cmax demonstrated similar findings to AUC as shown in Table 6. The GMRs of Cmax for baseline corrected EPA between OM3-MAG and OM3-TAG were 129.44 (95% CI: 114.60, 146.20), indicating an increase of 29% in the OM3-MAG when compared with OM3-TAG. The DHA levels were not significantly different between the OM3-MAG and OM3-TAG product groups. Significant differences were found in Tmax between OM3-MAG and OM3-TAG. The EPA and DHA baseline-adjusted Tmax were reached 5 h before with OM3-MAG compared to TAG (*p* < 0.05) a shown in Table 6.

In general, increases for EPA were observed in plasma but this was less true for DHA as is reported in Table 6. The OM3-MAG oil enabled to significantly increase the bioavailability (baseline-adjusted AUC and Cmax) of total EPA when compared to the OM3-TAG oil. Significant differences were also observed for the time to reach maximal concentration Tmax (earlier Tmax).

### 3.3. Clinical Trial C. Cystic Fibrosis Patients

#### 3.3.1. Clinical and Compliance Evaluation

Sixteen cystic fibrosis patients were screened and recruited, with an average age of 11.7 years. Group characteristics are shown in Table 7. One patient met the inclusion and exclusion criteria, but was not randomized due to expiration of the study product’s shelf life. All other subjects were 100% compliant with the study supplements for the duration of their participation in the study.

The provided supplements were well tolerated. Ten patients (5 in each product group) reported AEs (e.g., infective pulmonary exacerbation of cystic fibrosis, viral tonsillitis, pneumonia, *Pseudomonas* infection, impaired glucose tolerance test, haematochezia, urticaria), all of which were mild to moderate in nature and unrelated to study products. Serious AEs were recorded for two patients, both of which were in TAG products group. Subject characteristics and results for baseline analyses are shown in Table 7. At the beginning of the study, body weight average was 39.0 kg, and was not significantly different among groups at inclusion nor after 12 weeks. No significant differences were found among participants regarding lung function parameters (LCI, FEV1, FVC and MEF 25/75). Five patients had *Pseudomonas* colonization in the sputum samples at baseline (low to moderate amounts for OM3-MAG and low amounts for OM3-TAG). *Pseudomonas* colonization were reported for two patients only by the end of the study (moderate amounts for OM3-MAG and low amounts for OM3-TAG). Thus, the results are comparable in the two product groups.

Generally, a reduction in all nasal lavage inflammatory parameters was observed for patients on OM3-MAG, whereas for patients on OM3-TAG there was an elevation of these parameters during the study (Table 7). However, the difference between nasal lavage parameters was not statistically significant between the two products (*p* > 0.05).

#### 3.3.2. Incorporation of EPA and DHA in Erythrocytes and Plasma

The primary outcomes were relative and absolute levels of FA (EPA, DHA and EPA + DHA) in blood erythrocytes compared between OM3-MAG and OM3-TAG products (Table 1 and Table 3) given to cystic fibrosis patients for 84 days (Figure 3).

**Figure 6:**
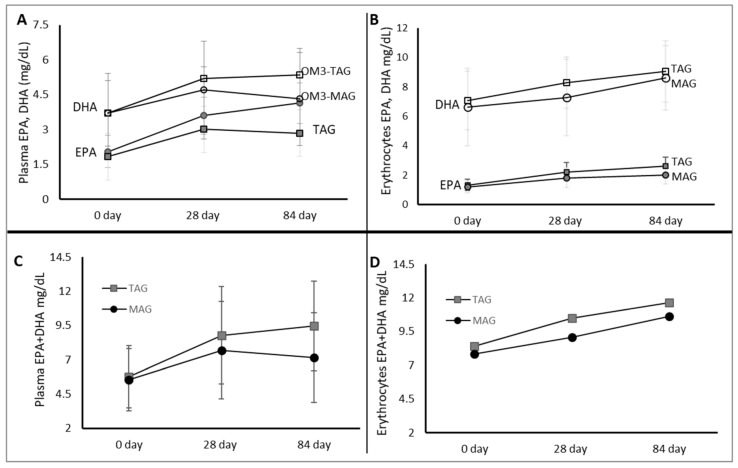
Clinical trial C. Accretion of DHA and EPA at 0, 28, and 84 day in plasma and erythrocytes (**A**,**B**) after MAG-enriched oil and TAG supplementation. EPA + DHA content per treatment group (**C**,**D**). Results are expressed in mg/dL).

At baseline, the EPA levels in erythrocytes for groups on OM3-TAG or OM3-MAG-enriched oil (1.33 and 1.19 mg/dL respectively), as well as DHA levels (7.07, 6.63 mg/dL) were not significantly different. Incorporation of EPA and DHA in erythrocytes at different time points is shown in Figure 6.

An effect modification was demonstrated at 84 days by both OM3-TAG and OM3-MAG oil treatments. The difference between OM3-TAG and OM3-MAG at 84 days for EPA and DHA was not significant (*p* = 0.09, 0.86, respectively). The assessment of accumulation of LC-PUFA in plasma showed that the EPA level at baseline was not significantly different between groups. Although the levels of plasma EPA were higher in the OM3-TAG group Figure 6A), we could not demonstrate a statistically significant difference (*p* = 0.06). Figure 6B shows that the level of EPA and DHA in the blood erythrocytes in the OM3-TAG group was not significantly higher over time than in the OM3-MAG group.

Figure 6A shows the levels of plasma EPA + DHA in the OM3-TAG group when compared with the OM3-MAG group. Same effect modification for EPA + DHA was observed through time in plasma and erythrocytes where OM3-MAG and OM3-TAG significantly increased the global absorption through time as shown in Figure 6C and 6D. The EPA + DHA levels were not significantly different between groups (in erythrocytes *p* = 0.52 and in plasma *p* = 0.13).

## 4. Discussion

Studies suggest that the EPA and DHA levels reached at steady state depend not only on the level of OM3 LC-PUFA supplemented but also on the nature of the dietary carrier used [15,19]. Our research here was designed to compare the oral bioavailability of OM3 LC-PUFA having different forms (i.e., MAG, ethyl ester, FFA and TAG), in different subject populations.

In the first postprandial cross-over study of healthy men, the OM3 LCPUFA were consumed as either MAG, FFA or ethyl esters, under a low fat diet. Our results demonstrated that the OM3-MAG and OM3-FFA have significantly higher bioavailability when compared with the ethyl ester carrier. Literature suggests that ethyl ester may be a poor substrate for pancreatic lipase (secreted in response to fat intake), with a subsequent decrease in the incorporation of FA into the mixed micelle [7,11,25,26]. The opposite can happen with OM3-FFA and OM3-MAG that are directly absorbed in the small intestine before entering circulatory system [15,19,24].

Different reports suggested clinical benefits according to the levels of OM3 LC-PUFA supplemented [5,7,27,43,44]. Like in the case of TAG levels reduction in blood after intake of high doses of EPA/DHA with up to 5 g per day [44]. For people with hypertriglyceridemia, dietary intake of fish/fish oil alone may not be enough to meet the OM3 recommended dose of 4g per day. The Lovaza prescribed ethyl ester form (840 mg of EPA+DHA) has been recommended for adults with severe hypertriglyceridemia. Mechanisms to reduce TAG levels are unknown but it has been suggested to reduce the hepatic production of TAG and very-low-density lipoprotein (VLDL) (lipogenesis) and increasing mitochondrial and peroxisomal β-oxidation in the liver [27,45]. In the ECLIPSE (Epanova^®^ Compared to Lovaza^®^ In a Pharmacokinetic Single-dose Evaluation I and II) studies, (Epanova^®^ versus Lovaza^®^, 4g) with overweight adults, better bioavailability was observed and lower levels of TAG for the FFA group compared with the ethyl ester group [7,29]. Higher levels of EPA + DHA in plasma of subjects on FFA after 14 days intake when compared with the ethyl ester group were also reported, similar to the findings in our trial. Previous comparisons have been made between ethyl ester and FFA with subjects under low fat consumption. Here, we have shown for the first time the comparison of OM3-ethyl ester versus the OM3-MAG to be a better carrier of OM3 LC-PUFA.

Previous results (while using lipase inhibitors) showed that the OM3-MAG enriched oil did not need an action of pancreatic lipase when compared with OM3-TAG [20,21]. Likewise, in the present study, the significantly higher levels of EPA+DHA in plasma lipids when OM3-FFA and OM3-MAG were ingested, strongly suggest that both enriched oils may also require minimal digestion prior to absorption as reported in our findings and/or literature [7,20,21,25,26,27,31,46,47]. Similar findings comparing OM3-ethyl esters with OM3-FFA, arginine salts or *sn*-2 MAG have reported lower incorporation of ethyl ester suggesting it to be due to either interferences or impairment in the LC-PUFA hydrolysis/absorption mechanism [7,26,27]. We have shown for the first time that the *sn*-1(3)-MAG is a better carrier than ethyl ester counterparts that contain bioactive OM3 LC-PUFA.

The same *sn*-1(3)-MAG carrier was also used in our second postprandial cross-over study for its comparison with OM3-TAG. The OM3 LC-PUFA were provided under low-fat conditions to obese or overweight subjects. Additionally, OM3 could be beneficial for obese population co-morbidities such as hypertriglyceridemia where individuals have increased circulation of lipoproteins and TAG. As mentioned before, the intake of OM3 LC-PUFA can be protective for this population since doses of 3-5 g/day have been used to treat hypertriglyceridemia, and have been shown to improve cardiovascular disease risk factors such as plasma TAG, blood pressure, and inflammation.

In the present study, the OM3-MAG carrier provided significantly higher bioavailability for EPA and not DHA compared with the OM3-TAG carrier. Our results here indicate that lipase activity is not required in the intestinal lumen, therefore OM3-MAG could have better uptake across enterocyte and eventually into the circulatory system, making OM3-MAG a more effective way to deliver the OM3 EPA to the body in order to prompt the metabolic benefits. Hence, we have observed that OM3 LC-PUFA supplementation could achieve the desired effect. Further benefits such as TAG reduction could be obtained with this carrier. Elevated levels of TAG in blood are characteristic of the hypertriglyceridemia condition and known risk factors for developing cardiovascular disease and increase the risk of pancreatitis [8,19].

The pancreas has a central function in digestion and glucose homeostasis, hence impaired excretion can result in maldigestion, malabsorption and malnutrition. In children, the most common cause of pancreatic insufficiency is cystic fibrosis where approximately 85% of patients show evidence of impaired lipid malabsorption at diagnosis and a high frequency of essential fatty acid deficiency [35,43].

Due to the reasons mentioned above, the *sn*-1(3)-MAG carrier was selected for the third trial to be compared with the TAG carrier, in cystic fibrosis patients known to be pancreatic insufficient. Our results demonstrated that both OM3-TAG and OM3-MAG forms allowed an accretion of OM3 LC-PUFA into the circulatory lipids from the erythrocytes and plasma blood compartments within three months. Several studies have suggested OM3-MAG as a good carrier of essential fatty acids, mainly for linoleic acid in cystic fibrosis. No specific information on positioning of FA (*sn*-2- versus *sn*-1(3)-MAG) was provided which makes drawing any conclusions more challenging [22,23,48,49]. Studies in cystic fibrosis children using MAG-linolenic acid have shown normalization of plasma, erythrocytes and platelet linolenic acid levels with or without the use of pancreatic lipases [22,23,50,51,52]. Other benefits such as a reduction in the concentration of circulating inflammatory markers in the plasma lipid profile and prostaglandins associated with bronchoconstriction and inflammation have also been shown [50,53]. Other cystic fibrosis studies report an increase in OM3 LCPUFA levels through the use of different carriers. Panchaud et al., (2006) reported higher levels of EPA (but no DHA) after feeding subjects a liquid PUFA mixture (0.2d EPA + 0.1g DHA) in a 12-month study. Significantly higher levels of EPA and DHA were reported in a 3-month study after feeding subjects an OM3 LC-PUFA blend (21.27% mmol EPA and 6.9% mmol DHA) [54]. Significant EPA increments were observed after feeding subjects 2 g capsule of fish oil (3.2 g EPA and 2.2 DHA) daily for 6 weeks. In a 60-day trial, feeding MAG-DHA (8 × 625 mg MAG-DHA) daily showed an increased DHA levels and a decreased AA ratio (AA/DHA) in erythrocytes [39,55]. Feeding DHA from algal oils has also resulted in significant increments of DHA in plasma, erythrocytes, and rectal DHA levels [56,57].

Previously, we reported OM3-MAG as a potent dietary carrier of DHA and EPA by providing superior bioavailability under impaired absorption conditions as compared to conventional dietary OM3-TAG. In the present study, the absorption and incorporation of OM3 LC-PUFA in circulatory lipids was significantly higher for both OM3-TAG and OM3-MAG after 84 days. These results strongly suggest that both carriers were well absorbed. The evolution of the concentration of OM3 LC-PUFA in both groups shows that dietary supplementation leads to a progressive increase of the concentration of EPA+DHA in circulatory lipids towards a steady state concentration. This may be due to the study span, or the relatively low OM3 LC-PUFA amounts fed to groups or a general health improvement secondary to observations of an increase in OM3 LC-PUFA final levels. A trend was only observed in the nasal lavage parameters and the lung function measured with no significant differences between treatments. Some cystic fibrosis trials reported no significant differences in lung function after OM3 supplementation [52,53,54,55]. OM3 LC-PUFA may also modulate inflammation through the reduction of plasma inflammatory cytokines (TNFa, IL-6), which are known to be potent promoters of inflammation and inflammatory markers such as CRP [55,56,57,58]. Unfortunately, unequal results are also observed for inflammatory markers and some have reported that after supplementation with OM3 LC-PUFA, IL-8 decreased significantly from a median [55,56,57,58,59].

In this study, due to the nature of the cystic fibrosis condition, complete removal of pancreatic intake from patients was not considered, nor was it necessary either to increase already standard doses. Those cystic fibrosis patients while increasing their dietary oil (i.e., fish oil) intake might at the same time change their intake of pancreatic enzymes due to symptoms such as eructation, stomach pain or diarrhea. Other studies have reported for participants with an increase in pancreatic enzyme doses except for the times when low levels of OM3 LC-PUFA supplementation were used.

There are several limitations to our three clinical studies. First, no compartmentalization analysis (e.g., cholesteryl esters, phospholipids, lipoproteins) of plasma was performed to better understand the lipid distribution in tissues together with metabolic changes after supplementation of OM3 enriched oils. Only the cystic fibrosis trial included both plasma and erythrocytes analysis, but not further phospholipid separation was performed. Second, we did not analyze the *cis*- *trans*- and positional isomers of the OM3 EPA and DHA that are present in natural OM3 sources and as blood metabolites. Third, in clinical trial B no measurements were performed on anthropometric or surrogate metabolic parameter to assess insulin resistance other than BMI, lack of overt dyslipidemia and lack of diabetes to establish whether subjects were metabolically stable. Fourth, different OM3 doses were used across clinical trials. Fifth, the improved bioavailability of OM3-MAG versus OM3-ethyl esters relies on single-dose comparison. Whether the significant improvement in bioavailability with OM3-MAG will translate into improved clinical efficacy over longer term studies is still yet to be determined. Future work would include longer and larger studies.

## 5. Conclusions

In conclusion, OM3-MAG and OM3-FFA display greater bioavailability than OM3-ethyl ester and should be considered when therapeutic high doses of OM3 are required. This might be even more important in genetic diseases with very high levels of TAG, requiring even higher effective amounts of OM3 [60]. The improved bioavailability observed with both carriers could translate into a larger TAG lowering effects and therefore provide greater beneficial effects on human health, particularly in cardiovascular disease.

In obese individuals, intake of nutritional doses of OM3 supplement MAG under low fat dietary conditions showed an increase in EPA absorption in acute dosing condition, but no significant changes in DHA when compared to TAG

In cystic fibrosis patients, a similar increase in EPA and DHA was found with both chronic dosing of either OM3-MAG and OM3-TAG. The initial low level of OM3 in this patient population suggest that OM3 supplementation should be considered [61]. For this population, the long-term intake of OM3 would seem beneficial, not only due to its potential therapeutic benefits but also preventatively in order to avoid deficiency and to improve their lipid profile. Further clinical studies with therapeutic doses of MAG are needed in patients to link clinical efficacy with improved OM3-MAG bioavailability.

## Figures and Tables

**Table 1:** Study groups and oil consumed per clinical trial.

Clinical Trial	Population	Age	Capsules	ACTIVE ARM		CONTROL ARM	
OM3			/Day	(mg/day)		(mg/day)	
				OM3-MAG		OM3-TAG	
				EPA	DHA	EPA	DHA
A. MAG	Normal weight	Adult	5	1655	1275		
Ethyl ester			4	1700	1380		
FFA			4	1748	1516		
B. MAG-TAG	Obese/overweight	Adult	9	560	362	774	564
C. MAG-TAG	Cystic fibrosis	4–10 years old	4	249	161	258	188
		11–18 years old	8	498	322	516	376

**Table 2:** Clinical trials inclusion and exclusion criteria.

OIL	Clinical Trial A OM3: MAG, Ethyl Ester. FFA	Clinical Trial B OM3: MAG, TAG	Clinical Trial C OM3: MAG, TAG
Inclusion	Healthy adults (18–65 years old) BMI normal to overweight range (18.5–29.9 kg/m^2^)	Overweight or obese adults (18–65 years old) BMI 25.0 to 34.9 kg/m^2^ (inclusive)	16 female/male patients diagnosed with CF and exocrine pancreatic insufficiency from 4–18 years old
Exclusion	- Medication affecting lipid metabolism - Pregnancy - Fish, or fish oil supplements within 2 months of study - Blood donation and participation in another clinical trial during the last month prior to the study - History of metabolic, cardiovascular, hypertriglyceridemia or type 2 diabetes mellitus diseases, diseases that could interfere with intestinal absorption.	- Medication affecting dietary fat absorption (i.e., Orlistat, Alli); interfering with OM3 uptake (i.e., blood thinning medication/anticoagulants) or lipid lowering (i.e., cholesterol/TAG lowering agent) - Consumption of OM3 supplements (greater than 500 mg/week) within 4 weeks of study - Following no fat or ultra-restrictive (less than 15%) low –fat diet - Allergic/adverse response to either study product - Having malabsorptive disorders, type 2 diabetes mellitus, hypertriglyceridemia	- Any change in CF prophylaxis regimen - Dietary supplements containing OM3 LCPUFA - Fish or fish oil supplements - Antibiotics within 4 weeks of study - Organ or hematological transplantation - Immunosuppressive therapy - Major complications of lung disease (including massive hemolysis, pneumothorax, or pleural effusion) within 8 weeks prior to baseline

**Table 3:** FA composition of capsules (in milligrams) before treatment.

	OM3-MAG	OM3-Ethyl Ester	OM3-FFA	OM3-MAG	OM3-TAG
Capsule	1 g	1 g	1 g	0.380 g	0.380 g
Total OM3- (% w/w) as:	MAG 90	Ethyl ester 92	FFA 91	MAG 92	TAG 90
EPA (mg)	331	425	437	62.25	64.5
DHA (mg)	255	345	379	40.25	47
SFA (mg)	<1.0	<1.0	<1.0	22.8	38
Other FA	400	230	184	277	268

**Table 4:** Clinical Trial A: EPA and DHA summary statistics (mean and SD), baseline-adjusted, for AUC over 24 h postprandial.

	OM3 Study Group			OM3 Study Group Comparison		
	MAG (*n* = 21)	FFA (*n* = 21)	Ethyl Ester (*n* = 23)	MAG-Ethyl Ester	FFA-Ethyl Ester	MAG-FFA
AUC0-24 h EPA	1486 ± 626	1496 ± 734	163 ± 251	3.41-fold *p* < 0.001	3.60-fold *p* < 0.001	0.95-fold, *p* = 0.60
C max EPA	143 ± 71	124 ± 48	17 ± 10	9.96-fold *p* < 0.001	8.58-fold *p* < 0.001	1.16-fold, *p* = 0.31
T max EPA	6.3 ± 4.4	5.2 ± 0.9	9.7 ± 6.5	3 h, *p* = 0.003	3 h, *p* < 0.001	0 h, *p* = 0.47
AUC0-24h DHA	1206 ± 600	1356 ± 676	562 ± 695	2.10-fold *p* < 0.001	2.34-fold, *p* < 0.001	0.90-fold, *p* = 0.49
C max DHA	113 ± 55	117 ± 39	44 ± 25	2.37-fold *p* < 0.001	2.48-fold *p* < 0.001	0.96-fold, *p* = 0.72
T max DHA	4.9 ± 0.9	5.7 ± 1.7	16.0 ± 8.3	7 h, *p* < 0.001	7 h, *p* < 0.001	0 h, *p* = 0.07

Results expressed in nmol·h/mL. Cmax in nmol/mL and T max in hours.

**Table 5:** Clinical Trial B. Study group characteristics.

	Study Groups	
	OM3-MAG (*n* = 29)	OM3-TAG (*n* = 30)
Age (years)	44.72 ± 9.83	44.10 ± 10.24
Male	11 (37.93%)	12 (40.00%)
Female	18 (62.07%)	18 (60.00%)
Weight (kg)	82.60 ± 12.86	82.12 ± 12.90
BMI (kg/m2)	29.64 ± 2.67	29.48 ± 2.77
Total cholesterol (mg/dl)	193.31 ± 29.08	194.30 ± 29.09
LDL-C (mg/dl)	117.41 ± 23.48	118.57 ± 23.92
HDL-C (mg/dl)	54.24 ± 10.91	53.97 ± 10.83
Triglycerides (mg/dl)	110.00 ± 37.59	110.53 ± 37.05

Values represent mean ± SD. MAG refers to enriched *sn*-1(3)-MAG oil.

**Table 6:** Clinical Trial B: EPA and DHA summary statistics, baseline-adjusted, for AUC over 24 h postprandial.

	Study Groups		Study Group Comparison
	OM3-MAG (*n* = 29)	OM3-TAG (*n* = 30)	OM3-MAG/OM3-TAG
AUC0-24h EPA	278 ± 108	236 ± 76.8	16% higher ^#^
C max EPA	18.2 ± 8.27	17.3 ± 6.8	29% higher ^#^
T max EPA	5 (3, 12) *	6 (5, 12)	5 vs. 6 *p* < 0.05
AUC0-24h DHA	173 ± 98	189 ± 105	11% lower ^##^
C max DHA	113 ± 55	117 ± 39	No difference
T max DHA	5 (3, 10) *	6 (4, 12)	5 vs. 6 *p* < 0.05

Results expressed in µG·h/mL. Cmax in ug/mL and T max in hours. AUC0-t, h·μg/mL. * hour, range. ^#^ Significant based on geometric least-square mean ratios MAG vs. TAG. ^##^ Not significantly different.

**Table 7:** Clinical Trial C: Study group characteristics/lung function parameters.

Study Groups		Age	Weight	LCI	FEV1	FVC	MEF	IL-1β	IL-6	IL-8	IP-10	NE
	DAY	Years	Kg					Log pg/mL				
OM3-TAG *n* = 8	0	11.8 ± 3.4	38.7 ± 13.6	11.75 ± 3.2	2.67 ± 1.27	2.02 ± 0.99	2.23 ± 1.64	0.21 ± 0.49	0.46 ± 1.64	1.79 ± 0.57	0.51 ± 0.61	2.36 ± 0.36
Mean ± SD	84		39.7 ± 13.6	12.29 ± 3.3	2.68 ± 1.25	2.05 ± 0.96	2.36 ± 1.67	0.25 ± 0.45	0.61 ± 1.67	1.93 ± 0.44	0.69 ± 0.58	2.45 ± 0.40
OM3-MAG *n* = 7	0	11.5 ± 3.6	39.3 ± 13.5	11.6 ± 3.6	2.71 ± 1.28	2.20 ± 1.22	1.76 ± 0.99	0.21 ± 0.28	0.67 ± 0.33	1.86 ± 0.55	0.81 ± 0.65	2.62 ± 0.35
Mean ± SD	84		40.04 ± 13.4	12.70 ± 2.7	2.77 ± 1.34	2.23 ± 1.21	1.73 ± 0.93	0.18 ± 0.44	0.61 ± 0.64	1.89 ± 0.59	0.67 ± 0.86	2.53 ± 0.60

Values represent mean ± SD. MAG refers to enriched *sn*-1(3)-MAG oil. LCI = lung clearance index, FEV1 = forced expiratory vital capacity, FVC = forced expiratory volume per second, MEF 25/75 = mean exploratory flow between 25% and 75% of vital capacity.

## Abbreviations

OM3	Omega-3 fatty acids
OM3-MAG	OM3-*sn*-1(3)-monoacylglycerol
FFA	free fatty acids
TAG	triacylglycerols
EPA	eicosapentaenoic acid
DHA	docosahexaenoic acid
LC-PUFAs	long chain polyunsaturated fatty acids
*sn*-2 MAG	*sn*-2 monoacylglycerol
FA	fatty acid
TC	total cholesterol
LDL-C	low-density lipoprotein-cholesterol
HDL-C	high-density lipoprotein-cholesterol
LCI	lung clearance index
FEV1	forced expiratory vital capacity
FVC	forced expiratory volume per second
MEF 25/75	mean exploratory flow between 25% and 75% of vital capacity
FAME	fatty acid methyl esters
AUC	area under the curve
GMR	geometric least-square means
LSMs	least square means
SAEs	serious adverse events
AEs	adverse events
TEAEs	treatment-emergent adverse events
GMRs	geometric least-square mean ratios
